# Draft Genome Sequence of *Veillonella parvula* HSIVP1, Isolated from the Human Small Intestine

**DOI:** 10.1128/genomeA.00977-13

**Published:** 2013-12-05

**Authors:** Bartholomeus van den Bogert, Jos Boekhorst, Eddy J. Smid, Erwin G. Zoetendal, Michiel Kleerebezem

**Affiliations:** Top Institute Food and Nutrition (TIFN), Wageningen, the Netherlandsa; Laboratory of Microbiology, Wageningen University, Wageningen, the Netherlandsb; Centre for Molecular and Biomolecular Informatics, Radboud University Medical Centre, Nijmegen, the Netherlandsc; NIZO Food Research B.V., Ede, the Netherlandsd; Laboratory of Food Microbiology, Wageningen University, Wageningen, the Netherlandse; Host-Microbe Interactomics Group, Wageningen University, Wageningen, the Netherlandsf

## Abstract

*Veillonella* species are frequently encountered commensals in the human small intestine. Here, we report the draft genome sequence of the first cultured representative from this ecosystem, *Veillonella parvula* strain HSIVP1. The genome is predicted to encode all the necessary enzymes required for the pathway involved in the conversion of lactate to propanoate.

## GENOME ANNOUNCEMENT

The genus *Veillonella* encompasses Gram-negative cocci that are commonly encountered in the human oral cavity (1, 2) and parts of the gastrointestinal tract, including the esophagus (3), stomach (4), and small intestine (5–7) (M. M. Leimena and B. van den Bogert, unpublished data). *Veillonella* spp. often co-occur with streptococci in these ecosystems, which is likely related to the capacity of *Veillonella* to effectively utilize lactate, derived from the fermentation of sugars by streptococci, as an energy source (8–10).

Using a selective cultivation approach, small intestine isolates closely related to *Veillonella parvula* were obtained from ileostoma effluent (11). The draft genome sequence of a representative isolate, *V. parvula* HSIVP1, was determined to investigate its metabolic capacity, with a special focus on lactate utilization.

Genomic DNA from *V. parvula* HSIVP1 was sequenced using 454 GS FLX (Roche) technology in combination with titanium chemistry, producing 168,415 reads of ~300 bp, and Illumina HiSeq 2000 technology, producing 10,356,186 paired reads of 50 bp, from 3-kb mate-pair libraries (GATC Biotech, Konstanz, Germany). Pyrosequencing reads were assembled using the Celera Assembler version 6.1 (http://sourceforge.net/apps/mediawiki/wgs-assembler/index.php?title=Main_Page) in 27 contigs. A pseudoassembly was constructed by placing the contigs in their likely order based on paired-read Illumina sequencing data using the SSPACE software version 1.1 (12) and a synteny comparison with the genome of a closely related strain, *V. parvula* DSM 2008 (GenBank accession no. NC_013520). The pseudoassembly was manually screened for inconsistencies using the Artemis comparison tool (13). The HSIVP1 genome was remarkably similar to that of *V. parvula* DSM 2008, displaying only minor chromosomal inversions (<5,000 bp) (B. van den Bogert, unpublished data). The final assembly of the *V. parvula* HSIVP1 genome contains 2,177,885 bp, with an average ~475-fold coverage and a G+C content of 38.51%, and the RAST server (14)-based annotation contains 2,014 predicted protein-coding genes.

The genes in the HSIVP1 genome were assigned to Clusters of Orthologous Groups (COG) (15) categories, using a BLASTp comparison with the COG database (NCBI, ftp://ftp.ncbi.nih.gov/pub/COG/COG) with an alignment *E* value cutoff of 10^-3^. As most *Veillonella* species cannot ferment carbohydrates (16), it is no surprise that the genome of HSIVP1 carries few genes (2.4%) assigned to carbohydrate transport and metabolism. A large fraction of the protein-coding genes was assigned to functions in energy production and conversion, which encompasses most of the genes known to be required for the conversion of lactate to propanoate. This pathway was completely encoded in the HSIVP1 genome and includes the characteristic methylmalonyl-coenzyme A (CoA) decarboxylase that generates a transmembrane electrochemical (Na^+^) gradient (17). This pathway is a critical component of the metabolic relationship in the small intestine ecosystem, where *Veillonella* is proposed to generate energy from the fermentation of lactate produced by (diet-derived) carbohydrate-fermenting lactic acid bacteria (e.g., *Streptococcus*). This proposed metabolic relationship is supported by the high level of expression of the lactate import permease and membrane-associated lactate conversion machinery of *Veillonella* in the small intestine (Leimena and van den Bogert, unpublished).

### Nucleotide sequence accession numbers.

This whole-genome shotgun project has been deposited at DDBJ/EMBL/GenBank under the accession no. ASKE00000000. The version described in this paper is version ASKE01000000.
